# Shwachman–Diamond syndrome: A case report

**DOI:** 10.1097/MD.0000000000039210

**Published:** 2024-09-06

**Authors:** Zumiao Liu, Qing Tang, Xiuqi Chen, Li Huang, Liancheng Lan, Zili Lv, Xia Yang, Qingwen Shan

**Affiliations:** a Department of Pediatrics, The First Affiliated Hospital of Guangxi Medical University, Nanning, China; b Department of Pathology, The First Affiliated Hospital of Guangxi Medical University, Nanning, China.

**Keywords:** clinical manifestation, *SBDS* gene mutation, Shwachman– Diamond syndrome

## Abstract

**Rationale:**

Shwachman–Diamond syndrome (SDS) is a rare autosomal recessive genetic disease, the diagnosis is a big challenge for clinician, as the clinical manifestations of the disease are diverse. Here, we report a girl who diagnosed with SDS with the symptoms of recurrent fever, elevated transaminase levels, and granulocytosis. The aspects of diagnosis and treatment were discussed and a literature review was conducted.

**Patient concerns:**

A 15-month-old girl admitted to our hospital because of recurrent fever, granulocytopenia, and elevated transaminase levels.

**Diagnosis and interventions:**

The compound heterozygous variant of Shwachman–Bodian–Diamond syndrome c.258 + 2T > C:p.84Cfs3 and c.96C > G:p.Y32* were detected after sequencing the blood samples from the patient and her parents. Finally, she was diagnosed with SDS and she was treated with compound glycyrrhizin, granulocyte-colony stimulating factor, and antibiotic in the case of co-infection.

**Outcomes:**

During the follow-up, her liver function showed the level of transaminases decreased and she rarely had infection after the age of 15 months although neutropenia is still present.

**Lessons:**

Patients with SDS lacks typical clinical symptoms, which presents a huge challenge for clinicians. Genetic testing techniques is playing an important role in the diagnosis of diseases. This patient without typical clinical manifestations such as exocrine pancreatic insufficiency and skeletal abnormality, we report this case aimed to strengthen the understanding of the disease.

## 1. Introduction

Shwachman–Diamond syndrome (SDS) is an inherited bone marrow failure syndrome characterized by 3 hallmark features: neutropenia, exocrine pancreatic insufficiency, and bony abnormalities.^[1]^ Progression and evolution of bone marrow disease remains a major cause of morbidity and mortality in these patients. The incidence of SDS is about 1/77,000 live births.^[2]^ Due to the financial difficulties, many families cannot afford too many tests, so early diagnosis and avoiding unnecessary tests are especially important. In this study, clinical and genetic analyses of a child with SDS and a literature review were conducted.

## 2. Case presentation

The patient was a 15-month-old girl who visited our hospital for further diagnosis and treatment. She was first admitted to an outside hospital at 3 months of age with the complaint of fever. Normal body temperature after oral ibuprofen. During a hospitalization at 6 months with the complaint of fever, she was found to have increased transaminase levels and was treated with antipyretics and a compound glycyrrhizin injection. Her symptoms improved, and liver function was decreased on recheck. At 8 months of age, she was admitted to an outside hospital with fever again the blood routine examination showed neutropenia (minimum 0.29 × 10^9^/L), during which she was treated with antipyretics and granulocyte-colony stimulating factor (G-CSF). The neutrophil count can rose to 4.17 × 10^9^/L but dropped below 0.5 × 10^9^/L again several months later. Because of recurrent fevers, hematologic abnormalities and liver function impairment, she came to our hospital for further treatment. Combined with the child’s genetic suggested Shwachman–Bodian–Diamond syndrome (*SBDS*) gene mutation, SDS was considered.

The patient was the second child of healthy unrelated parents. The child in this study was born in good condition, raised her head at 3 months of age, sat alone at 8 months of age, and now walks alone, and is the same developmental age as her peers. She has an older brother, now 12 years old, who is healthy and has no similar clinical manifestations.

**Figure 1.**
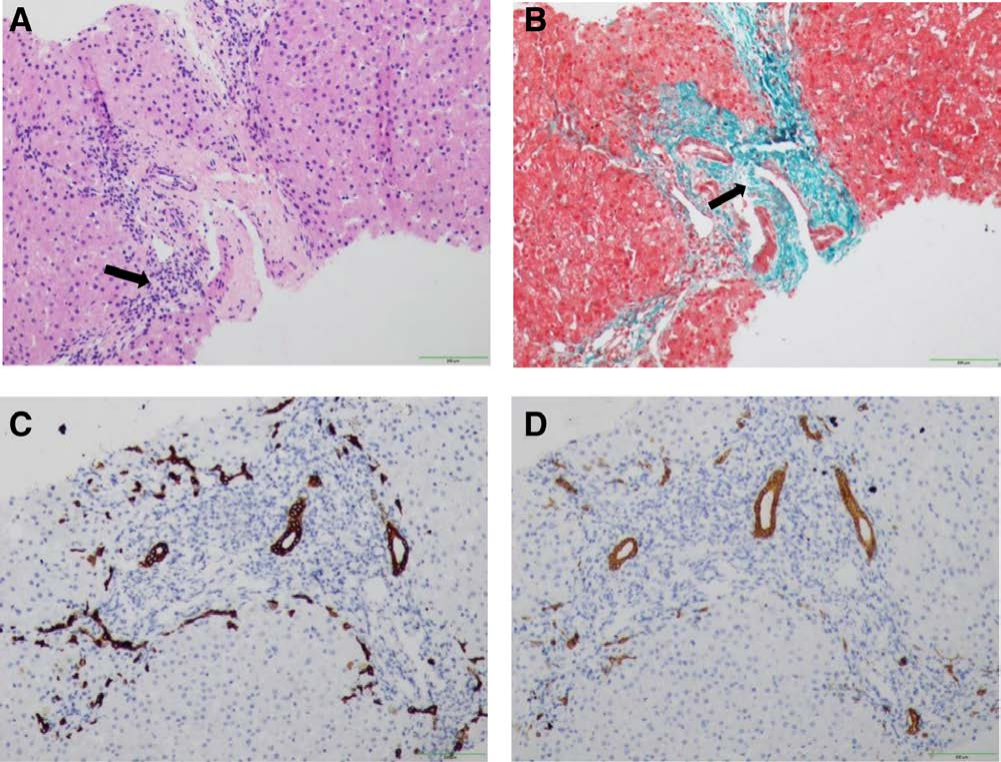
(A–D) Histological liver findings. (A) Hematoxylin and eosin staining of the liver tissue suggested the presence of mild edema of hepatocytes, punctate necrosis, and a small area of interfacial inflammation. (B) Collagen fiber Masson staining of the liver tissue (in green) suggested a small amount of collagen fiber hyperplasia. (C) IHC-CK19 showed hyperplasia of the interlobular bile duct. (D) IHC-CK7 showed hyperplasia of the interlobular bile duct.

At 15 months of age, our patient’s height was 78 cm (median − 1SD), her weight was 10.5 kg (median + 1SD). Several enlarged lymph nodes the size of soybeans could be palpated in the neck bilaterally. No skeletal malformations was observed. A swelling was seen in the umbilicus that could return. The patient’s white blood cell count and number of platelets were normal, the absolute neutrophil count was 0.81 × 10^9^ cells/L, and hemoglobin concentration was 117 g/L. Liver function showed that alanine aminotransferase (ALT) 335 U/L and aspartate aminotransferase (AST) 184 U/L, while total bile acid 17.9 µmol/L, and serum bilirubin and glutamyl aminotransferase were normal. The value of lipase and amylase were at a low normal level. A digestive system ultrasound include ultrasound of pancreas found no obvious abnormalities in the gallbladder, pancreas, or spleen. The patient’s parents refused abdomen CT scan because of the reasons of financial difficulties and radiation. Figure 1 shows the liver pathology results. Hepatocytes were mildly edematous with punctate necrosis, and a small area of interfacial inflammation could be seen (Fig. 1A). Masson staining showed a small amount of collagen fiber proliferation in the portal area (Fig. 1B). The portal area was slightly widened, with a small amount of fibrous tissue and juvenile interlobular bile duct hyperplasia (Fig. 1C and D) and more lymphocytic infiltration. The diagnosis was chronic hepatitis G2S2 with an ISHAK score of inflammation 5 and fibrosis 3.

### 2.1. Genetic testing

**Figure 2.**
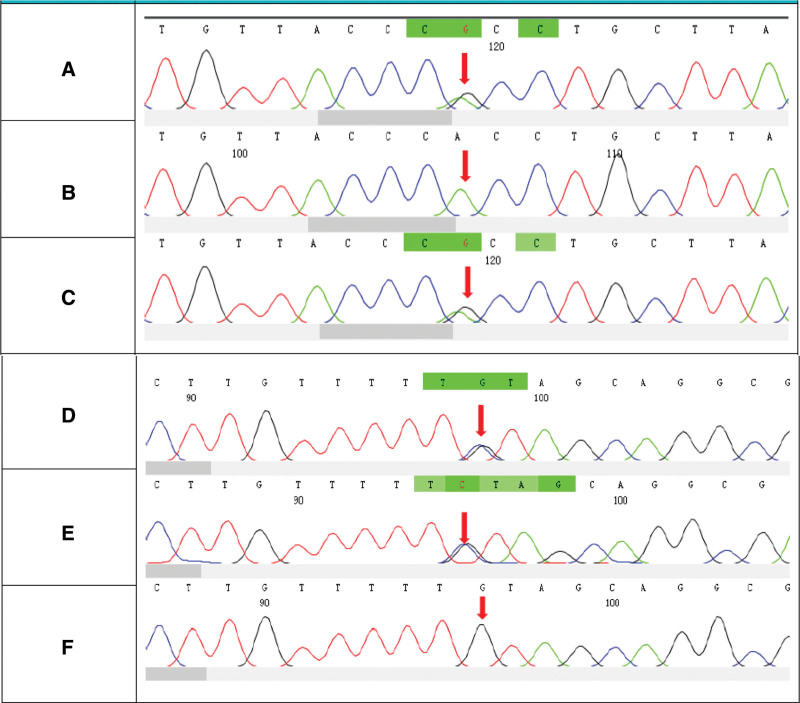
Sanger genetic testing results of the family. (a–c) The c.258 + 2T > C mutation in the *SBDS* gene. (d–f) The c.96C > G mutation in the *SBDS* gene. (a)The patient carries the c.258 + 2T > C mutation. (b) The genotype of this locus in her father is normal. (c) Her mother carries the c.258 + 2T > C mutation. (d) The patient carries the c.96C > G mutation. (e) Her father carries the c.96C > G mutation. (f) The genotype of this locus in her mother is normal.

The compound heterozygous variant of SBDS c.258 + 2T > C:p.84Cfs3 and c.96C > G:p.Y32* were detected after sequencing the blood samples from the patient and her parents (Fig. 2).

In her follow-up information, she was 94 cm (−1SD to median) in height and 13.6 kg (−1SD to median) in weight at 38 months. Since abnormal liver function (elevated ALT and AST levels) was found during the examination, the child was treated with oral liver protection drugs. The patient’s liver function showed a slightly elevated level. During the whole course of the disease, because the absolute neutrophil value was severely reduced, recombinant human G-CSF was injected to promote leukocyte treatment at 8 months old of the patient. The child’s routine blood examination showed that the absolute neutrophil value increased to 4.17 × 10^9^/L, but decreased to below 1.5 × 10^9^/L again several months later. As the child did not have repeated infections, G-CSF was not used again (Table 1).

**Table 1:** Laboratory findings from patient follow-up.

Age (months)	Leucocyte (×10^9^)	Hemoglobin (g/L)	Platelet (×10^9^)	Absolute value of neutrophils (×10^9^)	ALT (U/L)	AST (U/L)	Amylase (U/L)	Lipase (U/L)
15	9.05	113	280	1.31	77	111	-	-
16	10.19	113	233	1.18	92	173	-	-
18	9.21	126	246	0.4	78	147	-	-
20	7.51	123	213	1.01	246	136	-	-
23	6.08	124	209	0.36	62	121	-	-
25	6.36	120	262	0.72	93	92	9	19.2
27	6.96	122	291	0.45	71	70	11	7.5
29	6.95	120	213	0.85	87	101	-	-
38	5.46	127	158	0.34	63	83	8	9.6

*Notes*: Normal range of ALT: 7–45 U/L; normal range of AST: 13–40 U/L; normal range of serum amylase: 0–220 U/L; normal range of serum lipase: 0–60 U/L.

## 3. Discussion

We used PubMed as the primary database for finding articles on the subject. According to the case report statistics retrieved from the database, 152 cases of children had clear diagnoses and complete information were found. The clinical manifestations of patients with this disease are diverse. Some patients experience diarrhea, growth retardation, or recurrent infection as the first manifestation, while other patients have no clinical symptoms, and abnormal laboratory manifestations such as hemocytopenia are found on physical examination. The survival age of patients with SDS is related to the time of clinical presentation and the severity of the clinical manifestations. Among the patients reported in the literature, the oldest survivor is an adult male of approximately 60 years old.^[3]^

Among the 152 children with SDS-related clinical manifestations before 18 years old, 2 cases did not describe the patient’s sex. After collation of the data, 90 cases (59.2%) were male, and 60 (39.4%) were female, with a male-to-female incidence ratio of 1.5:1.

The reported clinical and physical manifestations are summarized in Table 2. The manifestations of pancreatic exocrine dysfunction include: increased stool frequency, high fecal fat content, decreased serum tryptase, amylase and lipase levels, radiographic changes, etc. The manifestations of hematologic involvement include: absolute neutrophil values, hemoglobin levels, or platelet values. The manifestations of skeletal involvement include: skeletal malformation, X-ray findings of long shaft diaphyseal dysplasia, etc. The manifestations of liver involvement include: liver enlargement, abnormal liver function, liver histological lesions, etc. The 2 most common clinical manifestations are exocrine function of the pancreas and hematologic. Moreover, 19 cases (12.5%) had hematologic malignancies, including 4 cases of acute myeloid leukemia (AML), 5 cases of MDS, 2 cases of lymphoma, 2 cases of aplastic anemia, and 1 case of MDS and AML.

**Table 2:** Summary of reported patient information.

Organ/system	n	%
Exocrine function of the pancreas	143	94%
Hematologic	148	97.3%
Skeleton	67	44%
Liver	62	40.7%

In the present case, the child had no diarrhea or feeding intolerance. The reason for this may be her young age, so she has not yet shown symptoms of pancreatic exocrine insufficiency. Several cases have shown that manifestations of pancreatic exocrine insufficiency can occur later in the course of the disease.^[2,4]^ Another reason is that only a small part of the pancreatic exocrine function is lost, but clinical symptoms of exocrine pancreatic insufficiency usually appear only when the loss of acinar volume exceeds 98%.^[5]^

In almost all affected children, persistent or intermittent neutropenia is a common presenting finding, often before the diagnosis of SDS is made.^[1]^ In this case, the routine blood examination of the child showed a decrease in the absolute value of the neutrophil granulocytes, and no abnormality was found in the classification and count examination of the peripheral blood white blood cells. No malignant hematologic disease was observed in the child. Because of the young age of the patient, it is still necessary to be vigilant about the possibility of leukemia and myelodysplastic syndrome.

Most patients with SDS come to hospital because of respiratory tract infection or unexplained fever. This may be because the absolute value of neutrophils in these child decreases, resulting in the body’s resistance to pathogenic microorganisms decreasing and becoming prone to infection.

The pathogenesis of liver involvement has not yet been clarified, but it has been proposed that the Fas pathway may accelerate hepatocyte apoptosis.^[6]^ Some studies have also suggested that the damage to liver function may be related to hepatocyte steatosis, which leads to impaired liver function. In some reported cases, liver pathological examination showed no specific lesions, different degrees of inflammatory cell infiltration, liver fibrosis, and hepatocyte steatosis.^[7,8]^ Liver abnormalities appear to manifest primarily in infancy with mild derangements in biochemical measurements of the liver, even severe liver involvement with massive hepatosplenomegaly and markedly abnormal liver biochemistry has been reported.^[9]^

Skeletal findings are symmetrical, can be widespread or localized, and are more marked in the lower limbs than the upper limbs. They vary in severity and location depending on the patient’s age. In infancy, the earliest radiographic findings are mild to moderate rib shortening and distal rib flaring or cupping.^[10]^ Published case series in SDS describe metaphy seal chondroplasia in 40 to 80% of patients and short stature in more than 50%.^[11]^ Most pediatric patients with SDS have an average birth weight in the 25th percentile, and the height and weight in more than 50% of these pediatric patients decrease below the 3rd percentile in the first year of life.^[12]^ This child has no skeletal deformity or short stature, which may be related to the absence of pancreatic exocrine insufficiency and no nutritional deficiencies.^[13,14]^

SDS is most commonly associated with biallelic mutations in the eponymous SBDS gene, named after the US physician Harry Shwachman, the British ophthalmologist Martin Bodian, and the American pediatrician Louis Diamond who reported the syndrome in 1964. In 2003, a positional cloning strategy identified biallelic mutations in the highly conserved SBDS gene in the majority of individuals with SDS.^[15]^ *SBDS* is expressed in all organs.^[16]^ The *SBDS* gene’s functions are unknown. One study showed that cells deficient in the *SBDS* protein are characterized by accelerated apoptosis and Fas hypersensitivity, suggesting that the protein might play an important role in Fas-mediated apoptosis.^[17]^ With the continuous advancement of genetic detection technology, in addition to the *SBDS* gene mutations that have been found in recent years, biallelic variants in DNAJC21, EFL 1, and SRP 54 have also been successively reported as causative gene mutations in SDS. Research has shown that *SBDS*, DNAJC21, EFL1, and SRP54 are involved in ribosome biogenesis: *SBDS*, through direct interaction with EFL1, promotes the release of the eukaryotic initiation factor 6 (eIF6) during ribosome maturation, DNAJC21 stabilizes the 80S ribosome, and SRP54 facilitates protein tracking. The authors suggest that their findings strengthen the postulate that SDS is a ribosomopathy.^[18]^

The most common mutation in the patients with SDS is the c.258 + 2T > C and c.183-184TA > CT composite heterozygous mutation. The second most common is the c.285 + 2T > C homozygous mutation. In this case, c.258 + 2T > C was detected in the patients with SDS and c.96C > G is a new mutation site that has not been reported in the literature. Current studies all show no obvious correlation between clinical phenotype and genotype in patients with SDS.^[18]^

The long-term treatment of SDS is multidisciplinary. No special treatment drugs were available for the patient. The main treatments options are alternative treatments and symptomatic treatments. For patients with exocrine pancreatic dysfunction, it is necessary to monitor pancreatic enzymes, such as amylase and lipase, oral tryptic enzyme replacement therapy, and fat-soluble vitamins (A, D, E, and K).^[19]^ Erythrocyte transfusion, platelet, and G-CSF may be considered for anemia, neutrophilia, and thrombocytopenia. For severe pancytopenia, those with MDS or AML should be considered for hematopoietic stem-cell transplantation.^[20]^ Our patient was treated with oral liver protection drugs due to elevated liver transaminase levels, and her liver function was reduced to normal, although there were repeated elevated values but no obvious aggravation. Liver lesions in SDS patients are generally mild, and many cases have reported that liver function impairment is most common in the first year of life and tends to return to normal^[10]^ after the age of 5 years.

## Author contributions

**Resources:** Qingwen Shan.

**Writing – original draft:** Zumiao Liu.

**Writing – review & editing:** Qing Tang, Xiuqi Chen, Li Huang, Zili Lv, Liancheng Lan, Xia Yang.
